# Tubeless percutaneous transhepatic one-step biliary fistulation combined with choledochoscopic lithotripsy for hepatolithiasis: a case report

**DOI:** 10.1055/a-2780-6059

**Published:** 2026-02-09

**Authors:** Canhua Zhu, Yanna Chen, Jiafen Xie, Junhua Cen, Guoying Wang, Qifeng Xu, Yanjun Luo

**Affiliations:** 1 117969 First Affiliated Hospital of Guangzhou Medical University, Guangzhou, China

Percutaneous transhepatic choledochoscopic lithotripsy (PTCSL) is one of the methods for hepatolithiasis. It is the standard practice to leave a transhepatic drainage tube after PTCSL, especially after percutaneous transhepatic one-step biliary fistulation (PTOBF). The drainage tube serves two purposes: to facilitate potential second-stage stone extraction or to be removed after tract maturation (typically 4–6 wk), providing that no residual stones are confirmed upon imaging 1 2 . However, we present a case in which external drainage was omitted following a PTOBF procedure, with only an internal biliary stent placed ( Video 1 ).

Tubeless percutaneous transhepatic one-step biliary fistulation combined with choledochoscopic lithotripsy with an indwelling biliary stent is successfully performed to treat hepatolithiasis. This procedure can achieve complete stone clearance in a single session without the need for percutaneous transhepatic drainage.Video 1

**Fig. 1:**
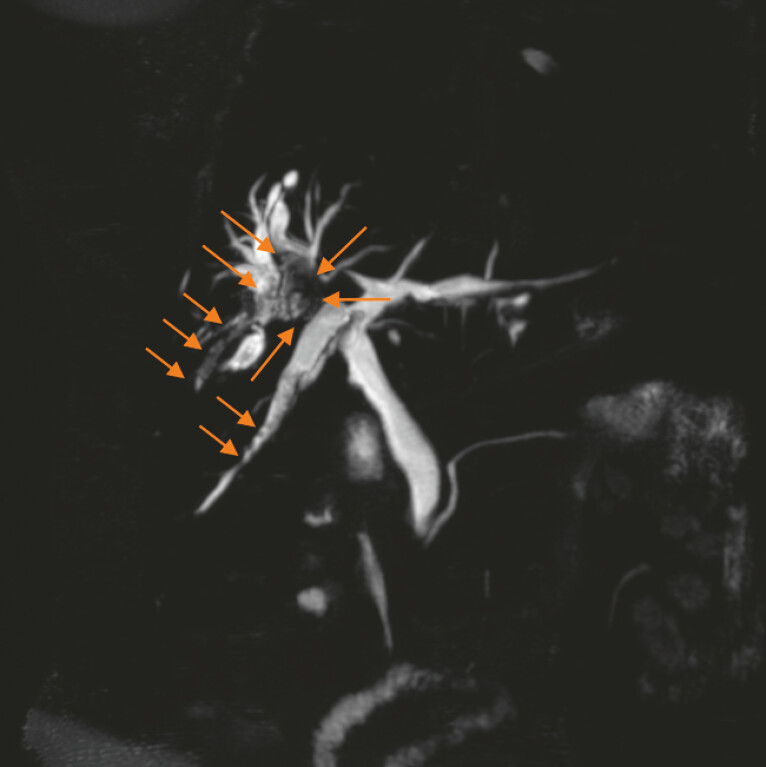
Magnetic resonance cholangiopancreatography showed hepatolithiasis in segments Ⅵ, Ⅷ, and the common hepatic duct (arrowheads).

A 58-year-old woman with a history of cholecystectomy was admitted for hepatolithiasis involving segments Ⅵ and Ⅷ and the common hepatic duct ( Fig. 1 ).

**Fig. 2:**
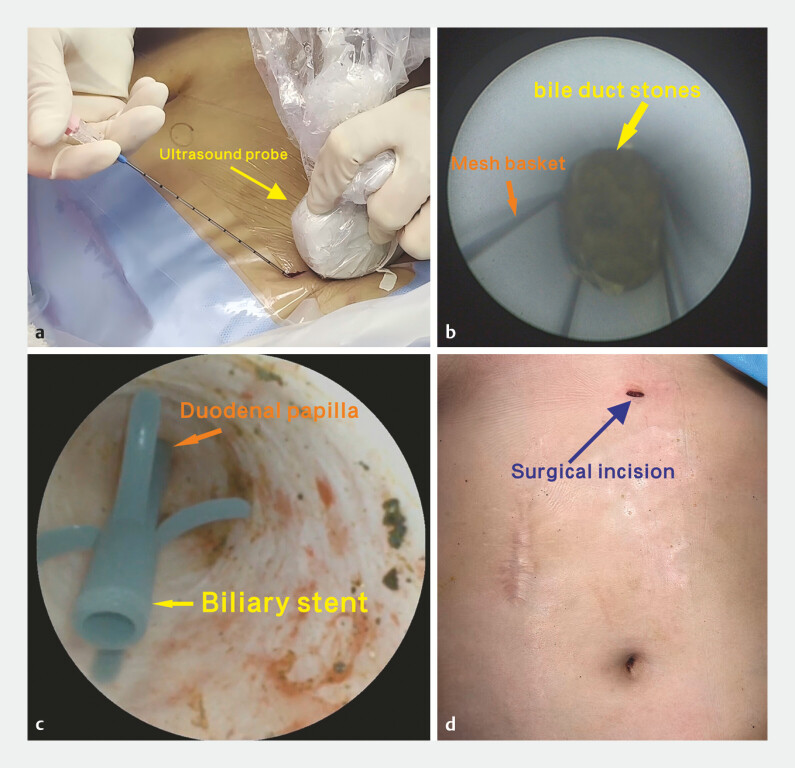
Percutaneous transhepatic one-step biliary fistulation combined with choledochoscopic lithotripsy for hepatolithiasis: **a** Ultrasound-guided puncture of the segment Ⅲ bile duct. **b** Stone extraction via a rigid choledochoscope and a mesh basket. **c** Placement of the biliary stent across the duodenal papilla. **d** The final appearance of the 5-mm incision with no external drainage tube placed.

The procedure was initiated under general anesthesia with an ultrasound-guided puncture of the segment Ⅲ bile duct ( Fig. 2 **a** ). A guidewire was then introduced into the bile duct through the puncture needle. Subsequently, the transhepatic tract was progressively dilated up to 14 Fr, and a sheath was left in place. A rigid choledochoscope was then advanced through the sheath into the bile duct, and stones were extracted using a mesh basket ( Fig. 2 **b** ). Complete stone clearance was confirmed by intraoperative ultrasound. A disposable flexible choledochoscope was advanced to the level of the duodenal papilla to confirm the absence of residual stones in the common bile duct. Finally, a guidewire was advanced through the papilla into the duodenum. After this, a biliary stent (TTSO-8.5-7; Cook Medical, Ireland) was deployed over the guidewire, positioning its distal end in the duodenum and proximal end in the common bile duct ( Fig. 2 **c** ).

Rigid choledochoscopy was repeated to confirm the absence of active bleeding within the transhepatic tract. The sheath was then removed, the skin incision was sutured, and the procedure was completed ( Fig. 2 **d** ). Postoperative abdominal computed tomography confirmed the absence of residual stones, demonstrated the appropriate stent position, and revealed no evidence of bile leakage. The patient had an uneventful postoperative recovery and was discharged on the second postoperative day.

This case demonstrates that a tubeless PTOBF combined with choledochoscopic lithotripsy can achieve complete stone clearance in a single session without the need for percutaneous transhepatic drainage.

Endoscopy_UCTN_Code_TTT_1AR_2AJ
